# The Study of Environmental Exposure of Mothers and Infants Impacted by Large-Scale Agriculture (SEMILLA): Description of the Aims and Methods of a Community-Based Birth Cohort Study

**DOI:** 10.3390/children11091045

**Published:** 2024-08-27

**Authors:** Alexis J. Handal, Fadya Orozco, Stephanie Montenegro, Nataly Cadena, Fabián Muñoz, Eileen Ramírez del Rio, Niko Kaciroti

**Affiliations:** 1 Department of Epidemiology, School of Public Health, University of Michigan, Ann Arbor, MI 48109-2029, USA; eilram@umich.edu; 2 School of Public Health, Universidad San Francisco de Quito USFQ (Ecuador), Quito 170157, Ecuador; 3 SEMILLA Project, Centro de Transferencia y Desarrollo de Tecnologías-USFQ, Quito 170157, Ecuador; 4 Visor Análisis Estadístico Cia. Ltda., Quito 170150, Ecuador; 5 Department of Pediatrics, School of Medicine, University of Michigan, Ann Arbor, MI 48109-2029, USA; nicola@med.umich.edu; 6 Department of Biostatistics, School of Public Health, University of Michigan, Ann Arbor, MI 48109-2029, USA

**Keywords:** community-engaged research, endocrine-disrupting chemicals, occupational epidemiology, pregnancy outcomes, Latin America, neurobehavioral development, birth cohort

## Abstract

Background/Objectives: Women of childbearing age not only reside in agricultural communities but also form an integral part of the agricultural labor force. Limited research investigates the impact of prenatal fungicide exposure on infant health, specifically ethylenebisdithiocarbamates and their toxic by-product, ethylenethiourea (ETU), particularly in occupational settings. This paper describes the background, aims, protocol, and baseline sample characteristics for the SEMILLA study, which investigates prenatal ETU exposure, neonatal thyroid function, infant growth, and neurobehavioral development in an agricultural region of Ecuador. Methods: This cohort study follows pregnant women and their infants up to 18 months of age, incorporating urinary biomarkers and survey data on ETU exposure and infant growth and neurodevelopmental measures. Data collection includes detailed questionnaires, scales, and physical examinations on maternal and infant health and development, as well as environmental factors. Descriptive statistics on key characteristics of the study population at baseline are presented. Results: SEMILLA enrolled 409 participants (72% enrollment rate): 111 agricultural workers (mostly floricultural), 149 non-agricultural workers, and 149 non-workers. Baseline characteristics show comparability between work sector groups, with some economic differences. Conclusions: SEMILLA will provide key evidence on prenatal fungicide exposure and infant development and encompass comprehensive multistage data collection procedures in pregnancy and infancy, focusing on structural and social determinants of health as well as individual-level chemical exposures. The community-based approach has proven essential, even amid challenges like the COVID-19 pandemic. The medium-term objective is to inform sustainable interventions promoting maternal and child health, with a long-term goal to reduce community exposures and improve worker health policies, particularly for women and pregnant workers.

## 1. Introduction and Background

Over 200 million children in lower- and middle-income countries (LMICs) are not reaching their full developmental potential [1]. Demand for increased food production worldwide and an expansion of export-driven floriculture and horticulture industries have increased the demand for fungicides. The global fungicide market is projected to reach US $30.9 billion by 2027 [2]. However, a significant gap exists in scientific knowledge about the neurobehavioral and developmental toxicity of fungicides. Importantly, women of childbearing age not only reside in agricultural communities impacted by large-scale agricultural production but also form an integral part of the agricultural industry labor force worldwide [3]. Little research has been conducted to assess the impact of exposure to fungicides during pregnancy on infant health outcomes, particularly in LMICs.

Ethylenebisdithiocarbamates, or EBDCs, are commonly used fungicides in agriculture, floriculture, and horticulture [4]. A few human studies have focused on exposure to fungicides, specifically EBDCs fungicides and their more toxic degradation product, ethylene thiourea (ETU), particularly in the occupational setting. Existing research on occupational ETU metabolite levels primarily focuses on male agricultural workers [5,6,7,8], with limited data on female workers. A handful of studies have documented increased ETU levels in women residing near agricultural fields or employed as agricultural workers [9,10,11]. However, studies are lacking that examine the association between prenatal ETU levels and thyroid dysfunction in pregnant women or their newborns, nor has research explored whether elevated prenatal ETU levels adversely impact infant growth and development. 

The Ecuadorian cut flower industry is representative of the growing agricultural export industry and provides an opportunity to evaluate occupational and environmental exposure to fungicides in agricultural communities and workers. Ecuador is the third-largest producer of cut flowers in the world. In Ecuador, cut flowers are a major export commodity, with US $937 million exported in 2021 (up ~7% from 2019), with approximately 43% exported to the United States [12]. Over half of the work force is female, mainly of reproductive age [13,14]. The cut flower industry has important environmental impacts [15]. Pesticide use in this industry is widespread, including the use of fungicides such as EBDCs. Further, this industry uses a large amount of water from the regions where farms are located [16]. A single flower farm uses, on average, around 237,000 L of water per hectare per month, and many of the farms lack proper filtration systems prior to water disposal back into the surrounding environment [17]. Residential communities are often located in close proximity to greenhouses. 

Our previous studies in a floricultural region of Ecuador identified associations between maternal employment and residence near large-scale industrial flower farms and delayed neurobehavioral developmental outcomes in young children [18,19,20,21]. Additionally, Handal and colleagues documented high levels of prenatal ETU metabolite levels among participants in a pilot study assessing the feasibility of quantifying prenatal pesticide levels in pregnant Ecuadorian women residing near or working in industrial floriculture greenhouses, suggesting potential exposure from both occupational and environmental sources in this region [22]. 

## 2. Objectives

The goal of the SEMILLA (Study of Environmental Exposure of Mothers and Infants Impacted by Large-Scale Agriculture) birth cohort study is to investigate and assess possible adverse effects of prenatal ETU exposure on newborn thyroid function, early childhood growth, and neurobehavioral development. 

To achieve this goal, SEMILLA aims to:

1. Measure associations between maternal levels of urinary ETU metabolites during pregnancy and infant growth and neurobehavioral development.
2. Determine whether the association between maternal urinary ETU metabolites during pregnancy and infant growth and neurodevelopmental outcomes is mediated by neonatal thyroid function.
3. Identify potential occupational and environmental predictors of elevated prenatal maternal urinary ETU levels.

Here, we present the methods and protocol for the SEMILLA study and describe the sociodemographic, maternal health, and economic characteristics of the cohort at baseline/enrollment.

## 3. Methods

### 3.1. Study Design

The SEMILLA study has a prospective longitudinal design carried out in a major agricultural region of Ecuador and led by researchers at the University of Michigan (AJH) and the Universidad San Francisco de Quito (FO). Birth cohort studies are an effective study design in epidemiological research as they are designed to prospectively observe the effects of early exposures at various time points throughout the developmental stages of a child [23,24]. This longitudinal birth cohort study follows pregnant women and their infants over approximately two years. 

### 3.2. Study Setting

**Figure 1:**
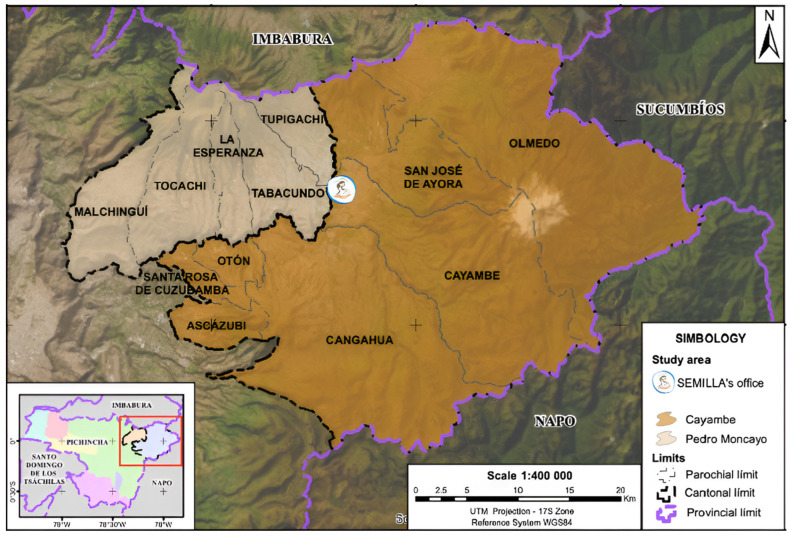
SEMILLA study region, Ecuador, 2018–2024.

The study area includes two specific cantons in the Pichincha province—Cayambe and Pedro Moncayo—which are located approximately 90 km northeast of the capital city of Quito in the northern highlands region of the country (Figure 1). The total population in the study area is estimated at approximately 118,967 inhabitants [25] and mainly concentrated in the Cayambe canton (72%), where 54.5% reside in rural areas [26]. In Pedro Moncayo, the percentage of rural inhabitants is higher (70%) [27]. This region was selected for several reasons: (1) this region has seen a dramatic increase over the past decade in greenhouse cut flower production and concentrate about half of the country’s flower production [28,29]; (2) the flower industry employs a large female labor force, mainly of reproductive age; and (3) this region was the focus of previous studies that inform the aims of SEMILLA [18,19,20,21,22].

### 3.3. Study Population

The study population includes pregnant women in the Cayambe and Pedro-Moncayo cantons. Inclusion criteria were established based on previous studies with the same population and included: being 18 years of age or older, 8–20 weeks gestation, living in the study area for at least one continuous year prior to recruitment, and expressing a decision to remain in the study area for at least one year after delivery.

Based on previous findings of high levels of ETU metabolite in both agricultural and non-agricultural workers [22], we sampled 3 groups of pregnant women as follows:

1. Floricultural and agricultural workers who are working for pay at enrollment.
2. Women who work in other types of work outside of the home for pay at enrollment.
3. Women who do not work outside of the home for pay at enrollment.

Including floricultural and agricultural workers allows the assessment of potential ETU exposure via occupation in the agricultural industry. Including non-agricultural workers and women who do not work outside of the home for pay (referred to as “non-workers” throughout this paper) allows the assessment of the potential occupational and environmental exposures occurring in the community beyond those associated with agricultural industry work, as well as important confounding and modifying variables such as stress and social support that may differ by occupational status. 

## 4. Recruitment and Enrollment

The intended sample comprised 420 participants, stratified by maternal occupational status at the time of enrollment, as described above. Initially, only floricultural workers were included in the first occupational category. However, due to the evolving COVID-19 situation and considering the increasing levels of labor insecurity, particularly in the flower industry, which was considered “non-essential”, as well as labor inactivity and female unemployment in the agricultural sector in general, other agricultural workers were included in that category as well [30]. 

Recruitment spanned 30 months (October 2019 to April 2022), with the COVID-19 pandemic and major social protests in the country forcing the shutdown of field activities at various periods during this time. Recruitment was carried out by a field team member designated as a “recruiter” who was supervised by the Technical Coordinator (SM). Outreach and recruitment strategies were diverse and included community engagement activities, liaison with health professionals and clinics in the region, as well as other key stakeholders (governmental and non-governmental), and promotion of the study through different means of communication, including radio, television, and social media. Collaboration with Ministry of Health personnel in the region facilitated dissemination through different spaces, including fifteen first-level health centers (nine in Cayambe and five in Pedro Moncayo) and the Basic Hospital in Cayambe [31]. 

Interested women underwent eligibility verification, including a confirmatory ultrasound (i.e., confirm a viable embryo within 8–20 weeks of gestation), which was subsidized by the project and performed by a collaborating local health care provider. If eligible, participants proceeded with the informed consent process.

## 5. Informed Consent

The Technical Coordinator conducted the consent process, reviewed consent forms with the potential participant, and answered any questions the woman may have before obtaining written informed consent for the woman’s participation and for the participation of her infant, once born into the study. The woman could be accompanied by her partner or a family member if she wished.

## 6. Follow-Up and Retention

The field team consists of the Technical Coordinator, a recruiter, and two trained community workers, otherwise known as the study interviewers, who are responsible for participant follow-up and interviews. Each community worker is responsible for tracking and evaluating their assigned participants and maintains regular contact with participants throughout study follow-up, both through regular phone calls and home visits. This approach is important as it builds trust with the participants and the study team and has served as a key retention strategy, especially given the complicated and lengthy nature of the data collection process and the sometimes sensitive nature of the data collected. Participants are provided incentives throughout the follow-up. The follow-up is ongoing. 

## 7. Data Collection Methods

SEMILLA is divided into two phases: 

Phase 1 (completed) was divided into two distinct periods of activities:

The first period, the pre-enrollment period, focused on project development and planning. This initial phase included establishing subcontracts with partner institutions, obtaining IRB approvals, securing project approval from the Ecuadorian Ministry of Public Health (MSP), hiring and training staff, engaging with community leaders and members, developing and adapting study instruments (with translation into Spanish and detailed review), digitizing all study instruments for electronic data collection, and preparing first drafts of study materials for pilot testing with pregnant women in the region. Field staff received training in human subject protection, interviewing, and data collection methods. 

The second period of Phase 1 involved the recruitment and enrollment of pregnant women into the study and the assessment of these women through the pregnancy. This period began in 2019; however, study activities were paused due to several social and political disruptions to the field work and the COVID-19 pandemic and resumed initially via phone contact in July 2020 due to continued pandemic-related restrictions and then fully resumed in September 2020 with a hybrid approach of in-person (biospecimens; height, weight, and blood pressure) and telephone data collection (questionnaires and surveys). Pilot testing of instruments was conducted on an ongoing basis during this period to ensure the validity and accuracy of study instruments and protocols, minimizing potential interviewer bias. 

As previously described, women were enrolled between 8 and 20 weeks gestation and provided up to three monthly first morning void (FMV) spot urine samples throughout their pregnancy, depending on their gestational age at enrollment. Urine samples were processed and stored at −20 °C until shipment to laboratory partners at the National Institute of Public Health of Quebec (the Centre de Toxicologie du Québec [32]) for analysis of ETU metabolite levels. At each prenatal visit, participants completed a comprehensive questionnaire (see Table 1; see Section 8), as well as a short questionnaire on interpersonal and emotional violence administered only at 32 weeks. During the prenatal visits, participants completed different assessments and screening instruments related to working memory (Digit Span test [33]), maternal depression (CESD-R [34,35]), and trauma (PTSD Checklist for DSM-5 [36]). Anemia status was assessed at each visit using a finger-prick hemoglobin screening method (namely, the HemoCue screening test [37,38]), and corrected for the altitude of the participant’s residence [39]. Maternal weight and height, as well as blood pressure, were collected at each visit by trained study team members. Maternal venous blood was collected up to three times during pregnancy, depending on her gestational age at enrollment, to assess thyroid levels. These samples were collected by either the Technical Coordinator (a medical doctor) or one of the study interviewers (an auxiliary nurse), both of whom were trained in this procedure. Subsequently, these samples were processed and transferred to a certified laboratory in Quito for analysis. Maternal hair and toenails were collected at each prenatal visit by team members trained in the appropriate collection methods and then labeled by the Technical Coordinator and stored at room temperature. 

The mode of application of the various study instruments depended on the context of the pandemic. During the peak pandemic time (July–November 2020), questionnaires and scales/instruments were administered over the phone due to the biosafety restrictions that were in place at that time. These instruments consisted of the following: baseline and pregnancy follow-up questionnaires, the Digit Span test (upon enrollment “baseline”), the CESD-R to assess depression in the mothers (upon enrollment “baseline”), the PTSD Checklist for the DSM-5 to screen for trauma in the mothers (second trimester), and a 24 h nutrition recall at 28 weeks gestation. Otherwise, all questionnaires and scales/instruments were administered in person to ensure privacy, comfort, and quality of the information collected.

Phase 2 involves following the mother–infant pair from delivery through 18 months postpartum. At the delivery visit (which could occur within two weeks after delivery, allowing for more flexibility and reducing any burden to the new mother), the mother was administered the HemoCue screening test, and newborn anthropometric measures were taken. A neonatal venous blood sample was also collected by a certified laboratory assistant with experience in neonatal blood collection at collaborating hospitals/clinics in the region. The neonatal blood sample was taken during an appointment scheduled for the mother by the study team. If the mother chose to stay at home, a certified laboratory technician would visit her at home to take the neonatal blood sample. During the delivery visit, the mother answered a questionnaire similar to the one administered during pregnancy, with additional questions regarding her delivery and postpartum experience, and completed three screening instruments (CESD-R and the Breastfeeding Efficacy Scale [40,41]), which were administered to the mother by telephone, respecting the mother’s recovery time at home with her newborn. The mother was also asked to provide a copy of her prenatal and delivery health cards as a backup for information not necessarily gathered during the interviews. All delivery visits have been completed. 

Then the mother–infant pair completes follow-up visits at the infant 3, 6, 9, 12, 15, and 18 months of age. Visits occur either over the phone or at the study center office in Cayambe, depending on what data are being collected. Generally, at each follow-up visit, mothers are interviewed about their working conditions, stress levels and social support, environmental exposures in home and work environments, interactions with their babies, lifestyle factors including dietary consumption patterns and nutrition, and socioeconomic factors. At each follow-up (3, 6, 9, 12, 15, and 18), the mother is administered the following instruments by phone: a mother/baby follow-up questionnaire (all time points), the Breastfeeding Self-Efficacy Scale (3 months), the Digit Span test (3 months and 18 months), the CESD-R depression screener (18 months), and a screening tool on family conflict, the Revised Conflict Tactics Scale (CTS-2) [42,43,44] (3 and 12 months). If the participant has time limitations or cannot complete the questionnaires or scales over the phone, they are given two options: (1) a home visit; or (2) at the study office, when the infant assessments are scheduled. A maternal 24 h nutritional recall (3 and 9 months) is conducted in person. 

At these follow-up visits, the infant undergoes developmental and growth assessments with trained study team members who involve the parents in the process whenever possible and appropriate. The infant assessments (growth, neurodevelopmental, vision, and nutritional) are conducted at the study office, providing a quiet space to conduct the infant testing. At all follow-up visits (3, 6, 9, 12, 15, and 18), the infant is screened for anemia status (HemoCue), and starting at 6 months, infant nutrition is also assessed (either by a 24 h food recall or by a food frequency questionnaire). Table 1 outlines the timing for the mother–infant interviews and assessments. 

Of note, the original protocol, as described above, follows the mother–infant pair until 18 months postpartum. However, the original study period was extended due to the pandemic situation, and as such, the team was able to extend mother–infant follow-up for a subset of participants whose infant would reach ~36 months (range: 24–39 months, depending on their time of enrollment) during the extension period. The specific data collection for this additional subset is outlined in Table 1. These interviews are ongoing.

## 8. Variables of Interest 

### 8.1. Outcome

The SEMILLA study focuses on assessing infant growth and neurodevelopmental outcomes. Infant growth measures (weight, length/height, and head and chest circumference) are measured at all time points. Standardized z-scores for anthropometric growth measures will be calculated using the WHO Anthro program (v. 2011) [45] to evaluate chronic and acute malnutrition. Neurodevelopmental assessment is measured using the Ages and Stages Questionnaire (ASQ-3) and the Bayley Scales of Infant and Toddler Development (BSID-III). The ASQ-3 screening test, designed for children aged 1 to 66 months, covers five broad developmental dimensions: communication, fine and gross motor skills, problem solving, and personal–social skills [46]. A continuous score is calculated for each age-specific questionnaire, with scores summarized for each developmental domain. The questionnaire is available in Spanish and has been previously used in this population [19,20]. The BSID-III, a norm-referenced (U.S. population) test [47], assesses several functional domains and consists of five scales: cognitive, motor, and language skills, and socio-emotional and adaptive behaviors. Raw scores are calculated for each subtest. Vision is also assessed as part of the infant’s neurodevelopment and has been found in previous studies to be impacted by prenatal chemical exposures [48,49,50], including a study by Handal and colleagues in the study region [19]. This outcome is evaluated by a trained ophthalmologist using standard procedures for pediatric populations, including visual acuity, ocular motility, red reflex, ocular surface examination, and refraction.

### 8.2. Exposure

The main study exposure is ETU metabolite levels during pregnancy. Exposure is assessed via two methods: prenatal first morning void (FMV) spot urine samples collected up to 3 times over the course of the pregnancy and questionnaire data on predictors of ETU exposure (work, home, environmental, food, and dietary habits). 

As previously described, urine samples were processed and stored at −20 °C until shipment to laboratory partners at the National Institute of Public Health of Quebec (the Centre de Toxicologie du Québec) for analysis of ETU metabolite levels. To quantify dilution, specific gravity (SG) was measured upon urine sample collection using a handheld digital refractometer (ATAGO, PAS-10S, Bellevue, WA, USA). Future analyses of urine ETU metabolite levels will consider both SG-adjusted and non-adjusted results, following established protocols and methods [51].

The Centre de Toxicologie du Québec (CTQ) has established protocols for the measurement of ETU. Briefly, chemical analysis methods involve enriching 100 μL of urine samples with labeled internal standard (ETU-d4), hydrolyzing with NaOH, derivatizing with pentafluorobenzyl bromide, and extracting with hexane. The extracts are then analyzed using Ultra Performance Liquid Chromatography (UPLC) with tandem mass spectrometry (MS/MS) in MRM mode. The detection limit is 0.033 μg/L, with intraday and interday precisions of 6.9% and 7.9%, respectively. The internal reference materials used to control the quality of the analyses will be at four levels (Near LOQ, Low, Med, and High) of in-house quality controls (QCs) prepared from an ETU standard source different from the calibration curve.

In accordance with approved IRB protocols, remaining urine samples not used for ETU analysis are stored at the University of Michigan for potential future analysis of additional chemicals.

### 8.3. Mediator

Neonatal thyroid function, as measured by levels of TSH and fT4, is considered a main mediator in this study. Neonatal thyroid function is assessed via neonatal venous blood collected at approximately 2 weeks postpartum by a certified laboratory assistant with experience in neonatal blood collection (see Section 7, Phase 2). 

### 8.4. Key Covariates

Neurobehavioral development is a complex process [1,52,53,54,55,56]. Data on key covariates addressing maternal and infant health and the social and physical environment of both the mother and the baby are collected through detailed maternal questionnaires and scales and through physical examination of the mother and infant. 

Key maternal variables: socioeconomic and demographic factors; working conditions and characteristics; environmental and social exposures in home and work environments; maternal stress, anxiety, and depression; social support and other social factors, including domestic/personal relationships; reproductive history; maternal health and lifestyle factors, including prenatal care and anemia status; iodine deficiency (urine) and manganese levels (hair); and COVID-19 pandemic-specific factors. 

Maternal nutrition is assessed via questions regarding nutrition and dietary consumption patterns in the maternal questionnaire and a 24 h nutrition recall applied at 28 weeks gestation and 3 and 9 months postpartum using an instrument that has been successfully used in other studies in Ecuador [57]. Food insecurity is assessed via questions generally based on the Food Insecurity Experience Scale (FIES) [58,59]. Participants are asked about food insecurity before the pandemic started (before March 2020) and after the pandemic started (after March 2020), to get a better understanding of food security issues within the pandemic context. 

Key infant variables: use of fungicides and other chemicals and environmental exposures [60] in and around the home; proximity of the home and daycare (if applicable) to a flower farm; infant exposure to irrigation water; crawling and mouthing behaviors; and health and social characteristics. 

Infant Nutrition and Current Health: Infant nutrition is a key indicator of neurodevelopment and growth [21,61]. At each infant visit, we administer a nutritional assessment (either a 24 h nutrition recall or a food frequency questionnaire, depending on the visit) to assess the micronutrients and macronutrients that the baby consumes at these age points as an additional approach to assessing predictors of child neurodevelopment and growth. Data on general infant health is also collected via the baby’s carnet, specifically birth weight and vaccination status. Infant anemia status is calculated based on the infant’s age and the altitude of his/her community of residence [62,63]. Questions on breastfeeding patterns and efficiency are also administered to the mother to assess early-infancy nutrition using the Breastfeeding Efficacy Scale [40,41].

Developmental stimulation at home and the interaction of the child with his/her family are important for child development [64]. We administer the Parenting and Child Development Questionnaire of Responsible Practice and Stimulation (CuPRE), a standardized and validated instrument that has been effectively used in similar populations in Latin America to measure support and stimulation available to the child at home [52,65].

## 9. Data Management Plan

Once all study instruments were pilot tested and finalized, they were converted to digital form using the Census and Survey Processing System (CSPro) [66] software system. The field team uses computers to collect data during the course of the study follow-up using CSPro. On an ongoing basis, as data are collected throughout the study period, it is saved on a cloud-based system, where the Ecuadorian data management team can then review it and check for errors or incomplete data. Data captured using CSPro is exported into STATA for cleaning. Data cleaning occurs for each data collection instrument after data collection is complete for each moment (e.g., baseline, pregnancy follow-ups, delivery, and mother/baby follow-ups) and includes running descriptive statistics (frequencies, means, etc.) and an assessment and possible recoding of open-ended variables (e.g., recoding of open-ended survey questions answers for the “Other” category). For each moment of data collection throughout the study period (e.g., baseline), the data management team creates a report that outlines the details about that particular database and a detailed codebook that describes the variables included in each STATA database. Once finalized, all databases and codebooks are shared with the University of Michigan team for future analysis. Storage of biospecimens (urine, serum, hair, and toenails) is located at the University of Michigan.

## 10. Study Size and Power Analysis

Based on previous cohort studies of this nature [10,67], our final sample consists of 409 women, including 111 agricultural workers, 149 non-agricultural workers, and 149 non-workers. Assuming a 20% attrition rate, and a two-sided Type I error α = 0.05, our sample provides sufficient power of 80% or higher for any pairwise comparison between work sector groups to detect a medium effect size of *d =* 0.4 *SD* for continuous outcomes and *OR =* 2.2 for binary outcomes. 

## 11. Statistical Methods/Approach

**Figure 2:**
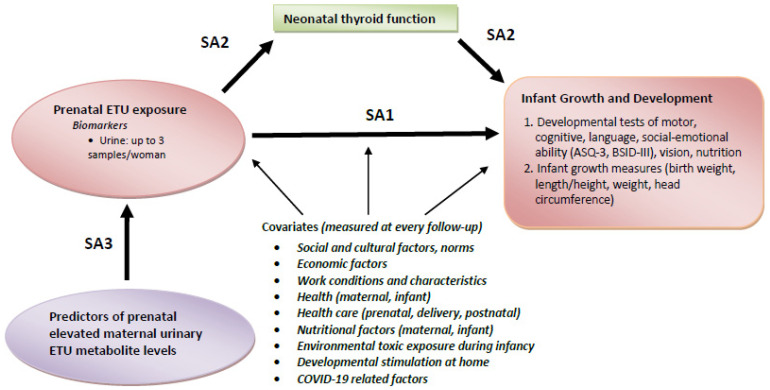
Conceptual model for assessing the association between maternal ethylenethiourea (ETU) levels, neonatal thyroid function, infant growth, and neurodevelopment. SEMILLA study. Cayambe, Ecuador. 2018–2024.

Figure 2 outlines our study aims (SA) and general analytic approach. SEMILLA’s recruitment and enrollment are concluded, and the team is presently conducting final mother/baby visits for the subset sample (24–39 months) and conducting data cleaning and management. 

The current paper presents descriptive statistics for the sociodemographic and maternal characteristics of the sample at baseline, by maternal work sector. All statistical analyses were performed with SAS 9.4. We compared the overall demographic characteristics of agricultural workers, non-agricultural workers, and non-workers using an analysis of variance (ANOVA) test for continuous variables and Fisher’s exact test for categorical variables. Next, we performed post-hoc tests (*t*-test for continuous variables and Fisher’s exact test for categorical variables) to assess pairwise differences for each variable by work sector. 

Once data collection is fully completed, we will perform initial analysis, including data reduction, transformation of skewed outcome variables when necessary, conducting descriptive statistics, and addressing potential outliers or errors. Despite efforts to minimize missing data, some loss-to-follow-up and incomplete measures are anticipated for this longitudinal cohort study. We will apply advanced techniques, such as “multiple imputations” [68], to impute the missing values. Our experienced team will perform sensitivity analyses to evaluate the robustness of the results under various missing-data scenarios [69]. 

The statistical models for testing the study aims outlined in Figure 2 will include both cross-sectional analysis and longitudinal analysis. Cross-sectional analysis will be the primary approach to assessing the association between prenatal ETU levels collected during each trimester of pregnancy and infant growth and developmental outcomes during the infant follow-up period, with longitudinal configuration incorporated whenever possible (variables that are standardized over time). Unadjusted bivariate analyses (Chi-square, *t*-test, correlations, and ANOVA) will examine the relationship between urinary ETU levels and infant growth and developmental outcomes. Crude associations with key covariates will be examined to identify potential confounding variables. Multivariable regression models will be used for adjusted analysis, controlling for potential confounders. For longitudinal analysis, mixed effect models with random intercept and slope will be employed for continuous outcomes to account for the within-subject correlation due to having repeated measures over time on the same subject. For categorical outcomes, Generalized Estimating Equations (GEEs) will be used with AR(1) autocorrelation structure, which will be allowed to vary by work group sector. We will also consider within-woman trends in ETU levels across pregnancy. In Aim 2, similar statistical modeling will determine the association between prenatal ETU levels and newborn thyroid function, as well as the link between newborn thyroid function and infant growth and development outcomes. Multivariate path analysis implemented in Mplus 8.10 (www.statmodel.com/) will be used to test whether newborn thyroid functioning mediates an association between maternal ETU metabolite levels during pregnancy and infant developmental outcomes. Multivariable regression models will be implemented to evaluate the predictors of prenatal elevated maternal ETU metabolite levels in Aim 3. For each model, the residuals will be evaluated to assess the model fit and determine whether the assumption of normality is satisfied. Appropriate transformation will be implemented if necessary to assure normality. All tests of statistical significance will be based on 2-sided tests at a significance level set at a *p*-value < 0.05.

## 12. Results

The SEMILLA study successfully enrolled 409 participants (72% of those eligible to participate). The sample includes 111 agricultural workers, with the majority working in floriculture, 149 non-agricultural workers, and 149 non-workers (women who were not working outside of the home for pay at the time of the baseline interview). 

Table 2 presents the socioeconomic demographics and general characteristics of the SEMILLA cohort study sample at baseline, by maternal work sector. The average maternal age for the sample was 27 years, with non-workers being slightly younger (26 years; *p*-value < 0.001). The gestational age at baseline was similar across all groups, averaging around 15 weeks. Perception of health status over the past 3 months prior to the baseline interview varied, with about half of participants reporting good health at the time of enrollment and about 40% reporting average/poor health at baseline, with non-workers reporting a greater proportion of average/poor health. 

Most participants resided in the Cayambe canton at the time of the baseline interview (75%), with similar proportions across all work sector groups. Non-agricultural workers and non-workers comprised the highest percentage residing in the Cayambe urban center. On average, participants reported living in the region for over a decade, though non-workers reported slightly less time (*p*-value: 0.0055). 

Housing characteristics were assessed. The majority of homes were reported as rented or obtained through services (53%). Agricultural workers reported a higher percentage of owning homes, while more non-workers reported renting or loaning homes. Almost all participants reported having electricity in their home. Participants were asked about the ownership of various appliances and other material goods in the home. As an overall marker of socioeconomic status, we created a scale for overall ownership of these appliances/materials. The material ownership scale includes YES/NO (scored as 1/0) answers for ownership of television, computer, internet (WiFi plan and cell phone plan), residential telephone, cellular telephone, smartphone, refrigerator, car, gas stove, electric stove, washing machine, and shower with hot water. ‘Don’t know’ and ‘No response’ answers were coded as 0 for the purposes of this scale. The range of this scale is (0, 13). On average, participants reported approximately 7 items on the material ownership scale, with non-workers reporting significantly less compared to the two worker groups (*p*-value: <0.001). 

Regarding residential environmental characteristics, the majority of participants reported having a toilet with a sewage system (80%), with significantly more non-agricultural workers reporting this characteristic. The majority of participants reported access to piped potable water for domestic use (96%) and for consumption (96%), with similar proportions across all work sector groups. Just under a quarter of the sample reported living near an irrigation ditch or open water canal, with an average distance of approximately 17 m. 

Household demographics were assessed. Participants reported approximately four household members, with similar proportions across all work sector groups. On average, participants reported approximately three household members over the age of 18 and roughly 2 household members younger than 18 years of age. Just over three-quarters of the sample reported living with their partner/husband (77%), with a larger percentage reported among agricultural workers. Between a quarter and a third of the sample reported living with their parents (21%) or other family members (34%). A majority reported living with children, with the largest proportion among agricultural workers (*p*-value: 0.0005). Finally, over three-quarters of the sample (77%) reported either being married or in a domestic partnership (free union) at the time of the baseline interview, with a slightly higher percentage among agricultural workers (83%; *p*-value: 0.0004). 

Participants were asked about their educational characteristics. Generally, almost all participants reported speaking Spanish or a mix of Spanish and Quechua at home. Maternal educational levels were assessed using categories used by the Ecuadorian government and converted to total years of education [70]. On average, participants reported a total of approximately 12 years of schooling. Levels in the sample varied significantly by occupational sector. Non-agricultural workers reported the highest number of years, followed by non-workers, with agricultural workers reporting the lowest number of years of schooling (*p*-value < 0.001). The partner’s education level followed a similar pattern. 

Participants were also asked about their financial characteristics. The average number of people working for income at home was approximately two, with non-workers reporting a lower average (*p*-value < 0.001). The average household income was $512, with approximately four people living off that income. Non-workers reported a significantly lower monthly household income ($404; *p*-value < 0.001), but with the same number of household members reliant on that income as the two worker groups. Husbands/partners were the primary income earners for all groups. Among those working for pay outside of the home, participants reported working on average approximately 39 h per week in the past week. Participants were asked about financial challenges, including difficulty meeting the costs of basic needs, rent, utilities, and education. We created a scale to assess financial strain and hardship. This scale includes YES/NO (scored as 1/0) answers for difficulty meeting the cost of food/other basic needs, housing payments, utilities, phone bills, clothing, education, health services, and other needs. ‘Don’t know’ and ‘No response’ answers were coded as 0 for the purposes of this scale. The range of this scale is (0, 8). Non-workers reported a significantly higher financial hardship scale score compared to agricultural and non-agricultural workers (*p*-value: 0.0054). 

Most participants identified as Mestiza (77%), though more agricultural workers identified as Indigenous compared to the other two work status groups. Finally, the majority of participants reported calm/good relationships in the home, though notably, more non-workers reported tense or violent relationships compared to the other two work status groups (5% versus 1% and 2%; *p*-value: 0.0218). 

## 13. Discussion

Although EBDCs are widely used in agricultural production and exposure to these products is increasing, representing a potential threat to children’s health, no human studies have focused specifically on how prenatal exposure to these fungicides or their metabolites (specifically ETU) affects newborn thyroid function, early childhood growth, or early childhood neurobehavioral development. This study represents a unique opportunity to investigate this question in women with previously documented high ETU levels—those working in large-scale agricultural industries and those residing close to these industries. Moreover, previous studies have assessed the impact of prenatal pesticide exposure on child neurobehavioral development, yet a few have focused on understanding mechanisms of action, particularly for fungicides. The SEMILLA cohort study examines both the impact of prenatal ETU exposure on newborn thyroid function and how poor thyroid function may mediate the impact of prenatal ETU exposure on infant growth and neurodevelopment. 

As presented in the results, the SEMILLA sample provides a good representation of working (agricultural and otherwise) and non-working participants—an important component for assessing varying levels of occupational and environmental fungicide exposure risk. Further, baseline characteristics demonstrate a socially and economically diverse study population. While the three work sector groups are generally comparable in sociodemographic aspects, there are notable economic differences, with non-workers experiencing greater financial hardship. This well-represented study population, especially in terms of work characteristics and key social, economic, environmental, and demographic factors, will be essential for SEMILLA to generate important evidence on the effects of prenatal ETU exposure on early child growth and development, as it will help elucidate the mechanisms of action by which this exposure affects maternal and newborn thyroid function and child growth and development while accounting for critical social, economic, and demographic characteristics. Further, because the SEMILLA study was carried out during the height of the COVID-19 pandemic, our findings will also help to document and understand the impact of the pandemic on the lives of pregnant women and their families in the region.

The SEMILLA study has several important strengths. The study is based on strong preliminary evidence and prior research, thus adding important evidence to the body of the literature. This cohort study is important because it addresses a question that has rarely been evaluated in human studies and takes advantage of a unique opportunity to investigate the question in a highly exposed population. As noted previously, epidemiologic studies that have focused on ETU exposure and thyroid function have been mainly among adult male agricultural workers [5,6,8]. A few studies have documented ETU levels in pregnancy, and the majority report urinary levels but do not assess neonatal thyroid function or other child health outcomes. This cohort study is the first in Ecuador to focus on this topic among pregnant workers and those living near large-scale industrial farms and one of only a handful of birth cohort studies conducted in Latin America, particularly for agricultural workers and in industrial agriculture contexts. Accurate characterization of female worker experiences, specifically those of the pregnant worker, has been a challenge in the areas of reproductive, environmental, and occupational epidemiology. SEMILLA will contribute important evidence to these areas of study. Furthermore, the SEMILLA study includes comprehensive multistage data collection procedures on various exposures (environmental, social, and structural) in pregnancy and infancy, as well as a strong focus on measuring and understanding key structural and social determinants of health beyond solely individual-level chemical or toxic exposures. Finally, the community-based approach, led by an interdisciplinary binational research team, has been essential for the successful implementation of the study, particularly during challenging periods such as the COVID-19 pandemic.

## 14. Conclusions

A large proportion of children in LMICs are believed to be falling short of reaching their optimal developmental potential [1]. Disruptions in early development can affect school readiness and performance and may have adverse social and economic consequences well into adulthood [71,72]. Environmental toxic exposure, poor nutrition, poverty, maternal stress and depression, and other social and economic factors contribute to the loss of developmental potential in young children [73]. As a larger number of women of reproductive age have joined the agricultural workforce across the world, with Latin America being no exception [3], more attention must be paid to the potential impacts of exposures on pregnancy health and the health of the infant.

The SEMILLA cohort study will contribute critical evidence to better understand the impact of environmental and occupational exposure to fungicides during pregnancy on infant growth and neurobehavioral development. This is a critically important area of research, not only in LMICs where fungicide use is widespread but also in high-income countries like the United States, where the use of fungicides is increasing and the scientific evidence of their health impacts continues to be limited. 

The goal of this study in the medium term is to inform appropriate and sustainable interventions to promote child health, neurodevelopment, and maternal health at the local and national levels and regionally across Latin America, especially during pregnancy, through the dissemination of results at all levels. The overarching long-term goal of SEMILLA is to reduce environmentally associated community exposures and improve worker health policies, particularly for women workers and pregnant workers.

## Figures and Tables

**Table 1:** Data collection procedures and measures for the SEMILLA study, Cayambe, Ecuador, 2018–2024.

	Phase 1			Phase 2							
	Enrollment (Baseline)	2nd Trimester	3rd Trimester	Delivery ^1^	3 Mths	6 Mths	9 Mths	12 Mths	15 Mths	18 Mths	~36 Mths ^2^
Maternal specific measures											
Maternal Questionnaire ^†^	✓	✓	✓	✓	✓	✓	✓	✓	✓	✓	✓
Weight/height	✓	✓	✓								
Blood pressure	✓	✓	✓								
Nutritional assessment—24 h recall		✓			✓		✓				
CESD-R	✓			✓						✓	
PTSD Checklist for DSM-5		✓									
Digit Span Test	✓			✓						✓	
Revised Conflict Tactics Scale—CTS2					✓			✓		✓	✓
Breastfeeding efficacy scale				✓	✓						
Infant specific measures											
ASQ-3					✓	✓	✓	✓	✓		
BSID-III								✓ *		✓	✓
Infant growth measures				✓	✓	✓	✓	✓	✓	✓	✓
Visual acuity						✓		✓ *		✓	
Nutritional assessment—24 h recall, food frequency questionnaire						✓	✓	✓	✓	✓	✓
Parenting and Child Development Questionnaire (CuPRE)							✓	✓	✓	✓	✓
Biological samples (mother and infant)											
Maternal urine (ETU; iodine levels at baseline only)	✓	✓	✓								
Maternal hair and toenails	✓	✓	✓								
Maternal venous blood (thyroid)	✓	✓	✓								
Maternal capillary blood (HemoCue-hemoglobin)	✓	✓	✓	✓							
Infant venous blood (thyroid)				✓							
Infant capillary blood (HemoCue-hemoglobin)					✓	✓	✓	✓	✓	✓	✓

^†^ Questionnaire data include: socioeconomic and demographic factors, work and home characteristics (social, environmental, and physical), social support and personal relationships, reproductive history, maternal health, and lifestyle factors; COVID-19 pandemic-specific factors; dietary consumption patterns, and infant environmental, social, and health characteristics. ^1^ Up to two weeks postpartum. ^2^ Study extension subsample followed until 24–39 months of age of the infant, depending on participant’s enrollment timeline. * Due to constraints in the timing of follow-up, some participants who enrolled later were only able to complete mother–infant follow-up for 12 months. In this case, the BSID-III and the vision test were administered to those infants.

**Table 2:** Socioeconomic demographics and general characteristics of the SEMILLA cohort study sample at baseline by maternal work sector. Cayambe, Ecuador. 2018–2024.

Characteristic	Total ^1^ (*N* = 409)	Agricultural Workers ^2^ (*N* = 111)	Non-Agricultural Workers (*N* = 149)	Non-Workers (*N* = 149)	*p*-Value ^3^
**Maternal age**					
M (SD)	27.2 (5.8)	28.7 (5.3) ^a^	27.5 (6.2) ^a^	25.8 (5.6) ^b^	<0.001
**Gestational age**					
M (SD)	15.3 (3.2)	15.1 (3.0) ^a,b^	14.9 (3.3) ^a^	15.8 (3.1) ^b^	0.0529
**Perception of health status *N* (%)**					0.1027
Excellent	33 (8.0)	7 (6.3) ^a^	16 (10.7) ^a^	10 (6.7) ^a^	
Good	208 (50.9)	61 (55.0) ^a^	81 (54.4) ^a^	66 (44.3) ^a^	
Average/poor	168 (41.1)	43 (38.7) ^a,b^	52 (34.9) ^a^	73 (49.0) ^b^	
**Current city of residence *N* (%)**					0.0983
Cayambe canton	162 (42.9)	46 (45.1) ^a^	61 (43.3) ^a^	55 (40.7) ^a^	
Cayambe canton (Cayambe urban center)	120 (31.7)	21 (20.6) ^a^	51 (36.2) ^b^	48 (35.6) ^b^	
Pedro Moncayo canton	42 (11.1)	15 (14.7) ^a^	13 (9.2) ^a^	14 (10.4) ^a^	
Pedro Moncayo canton (Tabacundo urban center)	54 (14.3)	20 (19.6) ^a^	16 (11.3) ^a^	18 (13.3) ^a^	
**Length of time living in the current city (years)**					
M (SD)	11.5 (11.6)	13.7 (12.4) ^a^	11.9 (11.7) ^a^	9.3 (10.6) ^b^	0.0055
**Status of current home *N* (%)**					<0.001
Owned by participant/her husband or partner	107 (26.2)	44 (39.6) ^a^	38 (25.5) ^b^	25 (16.9) ^b^	
Rented/loaned/through services	217 (53.0)	43 (38.8) ^a^	75 (50.3) ^a^	99 (66.3) ^b^	
Owned by parents	85 (20.8)	24 (21.6) ^a^	36 (24.2) ^a^	25 (16.8) ^a^	
**Length of time living in the current house (years)**					
M (SD)	6.8 (8.8)	8.7 (10.2) ^a^	7.2 (9.05) ^a^	5.0 (6.8) ^b^	0.0020
**Electricity in the home *N* (%)**					0.1151
No	3 (0.7)	0 (0.0) ^a^	0 (0.0) ^a^	3 (2.0) ^a^	
Yes	406 (99.3)	111 (100) ^a^	149 (100) ^a^	146 (98.0) ^a^	
**Material ownership scale**					
M (SD)	7.2 (1.9)	7.4 (1.5) ^a^	7.6 (1.9) ^a^	6.7 (2.0) ^b^	<0.001
**Type of hygienic service at home (toilet) *N (%)***					0.0342
None	3 (0.7)	1 (0.9) ^a^	1 (0.7) ^a^	1 (0.7) ^a^	
Toilet with septic tank	80 (19.6)	31 (27.9) ^a^	20 (13.4) ^b^	29 (19.4) ^a,b^	
Toilet with sewage system	325 (79.5)	79 (71.2) ^a^	128 (85.9) ^b^	118 (79.2) ^a,b^	
Does not know/remember	1 (0.2)	0 (0) ^a^	0 (0) ^a^	1 (0.7) ^a^	
**Type of water consumed at home *N* (%)**					0.7774
Open well	2 (0.5)	1 (0.9) ^a^	1 (0.7) ^a^	0 (0) ^a^	
Piped, non-potable (inside/outside of the home)	12 (2.9)	2 (1.8) ^a^	4 (2.6) ^a^	6 (4.0) ^a^	
Piped potable water	393 (96.1)	108 (97.3) ^a^	143 (96.0) ^a^	142 (95.3) ^a^	
Tank delivery by truck/bottled	2 (0.5)	0 (0) ^a^	1 (0.7) ^a^	1 (0.7) ^a^	
**Type of water for domestic use *N* (%)**					0.6846
Open well	2 (0.5)	1 (0.9) ^a^	1 (0.7) ^a^	0 (0) ^a^	
Piped, non-potable (inside/outside of the home)	14 (3.4)	2 (1.8) ^a^	6 (4.0) ^a^	6 (4.0) ^a^	
Piped potable water	392 (95.9)	108 (97.3) ^a^	142 (95.3) ^a^	142 (95.3) ^a^	
Tank delivery by truck/bottled	1 (0.2)	0 (0) ^a^	0 (0) ^a^	1 (0.7) ^a^	
**Distance to the irrigation ditch/** **water canal (meters)**					
M (SD)	16.7 (17.3)	24.2 (21.0) ^a^	14.7 (14.2) ^a,b^	12.9 (15.7) ^b^	0.0576
**Number of people that live at home**					
M (SD)	4.0 (2.1)	3.8 (1.5) ^a^	4.0 (2.2) ^a^	4.2 (2.3) ^a^	0.3771
**Number of people ≥ 18 that live at home**					
M (SD)	2.6 (1.3)	2.4 (1.2) ^a^	2.6 (1.3) ^a,b^	2.8 (1.4) ^b^	0.1233
**Number of people < 18 that live at home**					
M (SD)	1.4 (1.2)	1.4 (0.9) ^a^	1.3 (1.4) ^a^	1.4 (1.3) ^a^	0.8608
**Lives at home with partner/** **husband *N* (%)**					0.1182
No	95 (23.2)	18 (16.2) ^a^	39 (26.2) ^a^	38 (25.5) ^a^	
Yes	314 (76.8)	93 (83.8) ^a^	110 (73.8) ^a^	111 (74.5) ^a^	
**Lives at home with parents *N* (%)**					0.0079
No	325 (79.5)	99 (89.2) ^a^	114 (76.5) ^b^	112 (75.2) ^b^	
Yes	84 (20.5)	12 (10.8) ^a^	35 (23.5) ^b^	37 (24.8) ^b^	
**Lives at home with children *N* (%)**					0.0005
No	145 (35.4)	23 (20.7) ^a^	62 (41.6) ^b^	60 (40.3) ^b^	
Yes	264 (64.6)	88 (79.3) ^a^	87 (58.4) ^b^	89 (59.7) ^b^	
**Lives at home with other family members *N* (%)**					0.1014
No	272 (66.5)	82 (73.9) ^a^	99 (66.4) ^a,b^	91 (61.1) ^b^	
Yes	137 (33.5)	29 (26.1) ^a^	50 (33.6) ^a,b^	58 (38.9) ^b^	
**Marital status *N* (%)**					0.0004
Married	107 (26.2)	27 (24.3) ^a,b^	49 (32.9) ^a^	31 (20.8) ^b^	
Free union	209 (51.1)	65 (58.6) ^a^	59 (39.6) ^b^	85 (57.0) ^a^	
Separated/divorced/widowed	13 (3.1)	7 (6.3) ^a^	5 (3.3) ^a,b^	1 (0.7) ^b^	
Single	80 (19.6)	12 (10.8) ^a^	36 (24.2) ^b^	32 (21.5) ^b^	
**Language spoken at home *N* (%)**					0.0118
Spanish	396 (96.8)	103 (92.8) ^a^	147 (98.6) ^b^	146 (98.0) ^a,b^	
Quechua	1 (0.3)	0 (0) ^a^	1 (0.7) ^a^	0 (0) ^a^	
A mix of Spanish and Quechua	12 (2.9)	8 (7.2) ^a^	1 (0.7) ^b^	3 (2.0) ^a,b^	
**Mother’s educational level (years)**					
M (SD)	12.2 (3.7)	11.0 (3.3) ^a^	13.3 (3.9) ^b^	12.0 (3.4) ^c^	<0.001
**Spouse/partner educational level (years)**					
N	376	105	138	133	
M (SD)	11.7 (3.5)	11.0 (3.1) ^a^	12.5 (3.6) ^b^	11.4 (3.5) ^a^	0.0021
**The number of people who ** **currently work for income at home**					
N	396	107	140	149	
M (SD)	1.9 (1.0)	2.1 (0.9) ^a^	2.2 (1.0) ^a^	1.3 (0.8) ^b^	<0.001
**Total monthly income at home (US $)**					
N	364	99	125	140	
M (SD)	$512 (227)	$636 (201) ^a^	$533 (218) ^b^	$404 (200) ^c^	<0.001
**The number of people who live ** **on their monthly income at home**					
M (SD)	4.2 (3.3)	4.5 (4.6) ^a^	4.2 (2.9) ^a^	4.0 (2.4) ^a^	0.4866
**Primary person responsible for generating the majority of income at home *N* (%)**					<0.001
Participant	65 (15.9)	22 (19.8) ^a^	42 (28.2) ^a^	1 (0.7) ^b^	
Husband/partner	232 (56.7)	54 (48.7) ^a^	72 (48.3) ^a^	106 (71.1) ^b^	
Both earn the same	36 (8.8)	24 (21.6) ^a^	11 (7.4) ^b^	1 (0.7) ^c^	
Parents	42 (10.3)	4 (3.6) ^a^	18 (12.1) ^b^	20 (13.4) ^b^	
Other	22 (5.4)	7 (6.3) ^a^	5 (3.3) ^a^	10 (6.7) ^a^	
Not applicable (no one)	12 (2.9)	0 (0.0) ^a,b^	1 (0.7) ^a^	11 (7.4) ^b^	
**During the past week, the number ** **of weekly work hours**					
N	260	111	149	0	
M (SD)	38.5 (18.8)	39.4 (16.4) ^a^	37.8 (20.3) ^a^	-	0.4905
**Financial hardship scale**					
M (SD)	1.8 (1.8)	1.5 (1.6) ^a^	1.6 (1.8) ^a^	2.2 (1.7) ^b^	0.0054
**Mother’s self-reported ethnicity *N* (%)**					0.2405
Indigenous	88 (21.5)	31 (27.9) ^a^	29 (19.5) ^a^	28 (18.8) ^a^	
Mestiza	314 (76.8)	78 (70.3) ^a^	116 (77.8) ^a^	120 (80.5) ^a^	
Afro-Ecuadorian	3 (0.7)	1 (0.9) ^a^	1 (0.7) ^a^	1 (0.7) ^a^	
White	4 (1.0)	1 (0.9) ^a^	3 (2.0) ^a^	0 (0.0) ^a^	
**Partner’s ethnicity (reported by participant) *N* (%)**					0.4032
Indigenous	69 (16.9)	25 (22.5) ^a^	23 (15.4) ^a^	21 (14.1) ^a^	
Mestizo	298 (72.9)	80 (72.1) ^a^	109 (73.2) ^a^	109 (73.2) ^a^	
Afro-Ecuadorian	1 (0.2)	0 (0.0) ^a^	1 (0.7) ^a^	0 (0.0) ^a^	
White	6 (1.5)	1 (0.9) ^a^	3 (2.0) ^a^	2 (1.3) ^a^	
Does not know/does not remember	7 (1.7)	2 (1.8) ^a^	2 (1.3) ^a^	3 (2.0) ^a^	
Not applicable (no partner)	28 (6.9)	3 (2.7) ^a^	11 (7.4) ^a,b^	14 (9.4) ^b^	
**Relationship at home *N* (%)**					0.0218
Calm/good	383 (93.6)	101 (91.0) ^a^	144 (96.6) ^a^	138 (92.6) ^a^	
Indifferent	15 (3.7)	8 (7.2) ^a^	4 (2.7) ^a^	3 (2.0) ^a^	
Tense/violent	11 (2.7)	2 (1.8) ^a,b^	1 (0.7) ^a^	8 (5.4) ^b^	

^1^ Sample size for characteristics = total (*N* = 409); agricultural workers (*N* = 111); non-agricultural workers (*N* = 149); non-workers (*N* = 149), unless stated otherwise. ^2^ Majority floriculture workers (*N* = 102). ^3^ *p*-value reported is the overall test amongst the 3 work sector groups. Different superscripts (a, b, and c) indicate statistically significant differences between the work sector groups at *p* < 0.05 for levels of a characteristic.

## Data Availability

Once the SEMILLA data have been fully collected, analyzed, and main findings published, we will consider sharing data with qualified researchers, as our study population is vulnerable and care must be taken to protect participants. We would consider sharing the data under a data-sharing agreement that documents the users’ commitment to: (1) using the data for specifically defined research purposes agreed upon in advance, (2) providing Institutional IRB approval, (3) securing the data using appropriate computer technology, (4) acknowledgment of the source of data in any publication and citation of primary investigators, and (5) destroying or returning the data after analyses are completed.
